# A New Ant Species of the Genus *Tetramorium* Mayr, 1855 (Hymenoptera: Formicidae) from Saudi Arabia, with a Revised Key to the Arabian Species

**DOI:** 10.1371/journal.pone.0030811

**Published:** 2012-02-28

**Authors:** Mostafa R. Sharaf, Abdulrahman S. Aldawood, Brian Taylor

**Affiliations:** 1 Plant Protection Department, College of Food and Agricultural Sciences, King Saud University, Riyadh, Kingdom of Saudi Arabia; 2 Independent Researcher, Nottingham, United Kingdom; University of Arizona, United States of America

## Abstract

*Tetramorium amalae* sp. n. is described and illustrated from Saudi Arabia based on two worker caste specimens collected in Al Bahah region. The new species belongs to the *T. shilohense* group and appears to be closely related to *T. dysderke* Bolton from Nigeria. *T. amalae* is distinguished by having well-developed frontal carinae, smaller eyes, greater head length and width, greater pronotal width, and the petiole node is longer than broad. *Tetramorium latinode* Collingwood & Agosti is recorded for the first time from Saudi Arabia and for only the second time since the original description. The worker caste of *T. latinode* is redescribed and illustrated using scanning electron micrographs to facilitate recognition and the gyne is described for the first time with observations given on species relationships, biology and habitat. A revised key to the nineteen *Tetramorium* species recorded from Arabian Peninsula based on worker castes is provided. *Tetramorium bicarinatum* (Nylander) is recorded for the first time from Saudi Arabia. It is suggested that *T. amalae* and *T. latinode* are endemic to the Arabian Peninsula.

## Introduction

The ant genus *Tetramorium* is one of the largest hyperdiverse ant genera in the subfamily Myrmicinae. It contains more than 400 species and subspecies worldwide [1] and is distributed through the tropics and temperate regions [2]. Most *Tetramorium* species nest in decaying wood, leaf-litter, or directly into the soil [3] with many Arabian species nesting into open hard-packed earth under stones. Other species are known to be arboreal or termitolestic in Africa [3]. The world *Tetramorium* fauna was comprehensively revised for all zoogeographical regions except the Palaearctic region by Bolton [3]–[7] with additions by Radchenko [8], [9] for the former Soviet Republics, Hita Garcia *et al.* on some species-groups of the Afrotropical [10]–[12] and Malagasy [13] areas, Yamane & Jaitrong [14] on Laotian species, also Csösz *et al.* [15] and Csösz & Schulz [16] on Palaearctic species.

Within the subfamily Myrmicinae, almost all workers of the genus *Tetramorium* can be easily recognized by the following combination of characters [17]: lateral portion of clypeus raised into a sharp ridge or shield wall on each side, in front of the antennal insertions; sting with an apicodorsal lamellate appendage projecting from the shaft; either median clypeal carina or median cephalic carina usually present, or both present; palp formula predominantly 4, 3; mandibles armed with three or four teeth apically, followed by a variable number of denticles; antennae 11- or 12-segmented with a 3-segmented club; and with the metanotal groove usually impressed.

Bolton [3] recognized 19 species groups of *Tetramorium*, and in this paper we treat the species from the Arabian Peninsula (referred to as “Arabia” hereafter) as being in his *T.*s*hilohense* and *squaminode* groups. The *T. shilohense* group is distinguished by the following combination of characters: antennae with 12 segments; mandibles usually sculptured; frontal carina varying from strongly developed to absent; antennal scrobes from moderately developed to absent; eyes small to minute with maximum diameter less than 0.17×HW; and the propodeum armed with a pair of spines or teeth. The *T. squaminode* group is distinguished by the following combination of characters: antennae with 12 segments; anterior clypeal margin usually undented medially; frontal carina strongly developed, reaching back almost to posterior margin of head; antennal scrobes present; petiole squamiform, much higher than long in profile and much broader than long in dorsal view; postpetiole usually rounded nodiform; and sculpture predominantly absent from the petiole and postpetiole.

Little is known regarding the genus *Tetramorium* in Arabia as a whole. The present knowledge of these species is available in only two publications summarizing the taxa known from Saudi Arabia [18] and Arabia [19]. For Saudi Arabia, thirteen species have been recorded [18] two of which were described as new, *T. jizani* Collingwood from Fayfa and Abu Arish and *T. juba* Collingwood from Al Kharj and Al Kola. Sixteen species were listed and keyed for Arabia [19] and two additional species were described from Yemen, *T. latinode* from Mabar and *T. yemene* from Sid el Feyhn. A new species, *T. hirsutum* Collingwood & van Harten [20] was described from Yemen based on workers and queens. Three tramp species, *T. lanuginosum* Mayr, *T. simillimum* (F. Smith) and *T. caldarium* (Roger) were recorded from Socotra Archipelago [21].

*T. latinode* was described from a single worker. The original description is not adequate to distinguish the species, not even indicating the body colour, a character useful to distinguish it from *T. squaminode* Santschi, its congener. A comprehensive redescription of *T. latinode* using scanning electron micrographs is presented here.

In the present study, a new species of the genus, *T. amalae* is described from Saudi Arabia. General information on the habitat and affinities of the new species are given. The tramp species *T. bicarinatum* is recorded for the first time in Saudi Arabia. A revised key to the known *Tetramorium* species of Arabia is given, although it is our intention to produce a more comprehensive version when a full set of modern images can be completed and, where necessary, type material examined.

## Materials and Methods

### Measurements and indices

Measurements in mm and indices are as follows:

TL = Total Length; the outstretched length of the ant from the mandibular apex to the gastral apex. Although somewhat unreliable, due to shrinkage of the often soft gaster, this often is useful for sorting specimens.
HW = Head Width; the maximum width of the head behind the eyes measured in full face view.
HL = Head Length; the maximum length of the head, excluding the mandibles, measured in full face view.
CI = Cephalic Index (HW×100/HL).
SL = Scape Length, excluding basal condyle and neck.
SI = Scape Index (SL×100/HW).
EL = Eye Length; the maximum diameter of the eye.
ML = Mesosoma Length; the length of the mesosoma in lateral view, from the point at which the pronotum meets the cervical shield to the posterior base of the propodeal lobes or teeth (also known as “Weber's length”).
PW = Pronotal width, maximum width in dorsal view.
PL = Petiole Length; the maximum length measured in dorsal view, from the anterior margin to the posterior margin.
PTW = Petiole Width; maximum width measured in dorsal view.
PPL = Postpetiole Length; maximum length measured in dorsal view.
PPW = Postpetiole Width; maximum width measured in dorsal view.

All measurements are in millimeters and follow standard measurements [3], [13]. As absolute sizes are known to vary within and between samples of some ant species, indices, such as CI, often are more reliable for separating species with otherwise similar morphology and characters.

No specific permits were required for the described field studies or for the surveyed locations which are not privately-owned or protected in any way or do not have endangered or protected species.

### Nomenclatural Acts

The electronic version of this document does not represent a published work according to the International Code of Zoological Nomenclature (ICZN), and hence the nomenclatural acts contained in the electronic version are not available under that Code from the electronic edition. Therefore, a separate edition of this document was produced by a method that assures numerous identical and durable copies, and those copies were simultaneously obtainable (from the publication date noted on the first page of this article) for the purpose of providing a public and permanent scientific record, in accordance with Article 8.1 of the Code. The separate print-only edition is available on request from PLoS by sending a request to PLoS ONE, 1160 Battery Street, Suite 100, San Francisco, CA 94111, USA along with a check for $10 (to cover printing and postage) payable to “Public Library of Science”.

In addition, this published work and the nomenclatural acts it contains have been registered in ZooBank, the proposed online registration system for the ICZN. The ZooBank LSIDs (Life Science Identifiers) can be resolved and the associated information viewed through any standard web browser by appending the LSID to the prefix “http://zoobank.org/”. The LSID for this publication is:urn:lsid:zoobank.org:pub:63028708-CD01-4126-BF88-43E3800CB8A4

## Results

### *Tetramorium amalae* Sharaf & Aldawood n. sp

urn:lsid:zoobank.org:act:9BEC41D6-593C-4D65-947D-B6F19A8F5113

#### Holotype worker (Figs. 1, 2, 3, 4, 5, 6, 7, 8)

**Figure 1:**
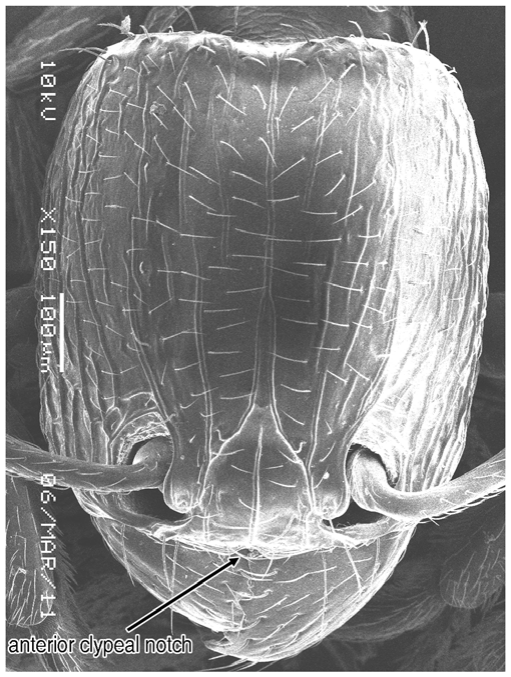
Scanning Electron Micrograph of *Tetramorium amalae* sp. n. holotype worker, head in full-face view.

**Figure 2:**
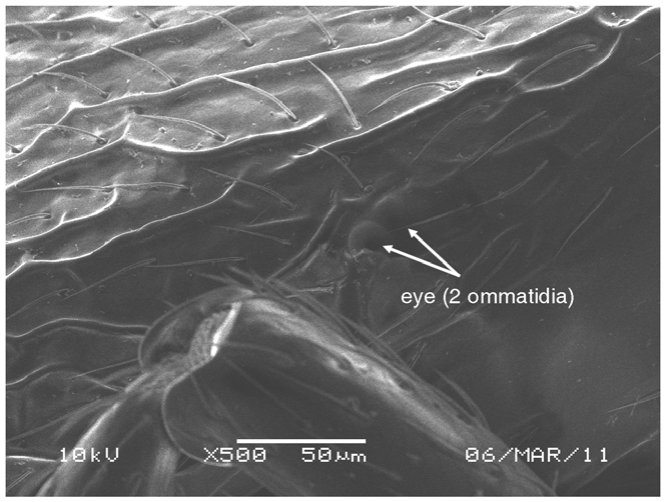
Scanning Electron Micrograph of *Tetramorium amalae* sp. n. holotype worker, eye.

**Figure 3:**
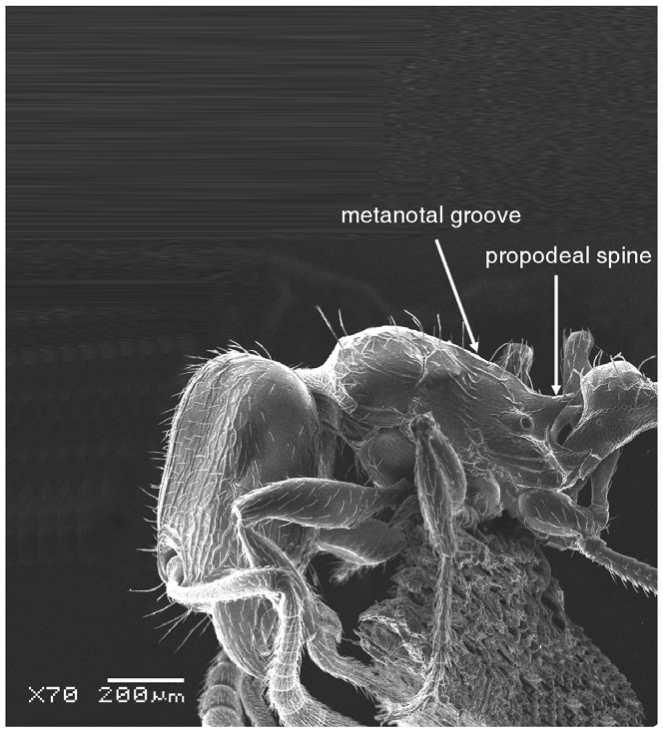
Scanning Electron Micrograph of *Tetramorium amalae* sp. n. holotype worker, body in profile.

**Figure 4:**
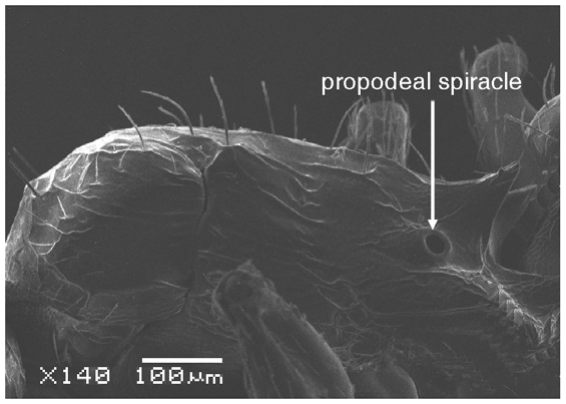
Scanning Electron Micrograph of *Tetramorium amalae* sp. n. holotype worker, mesosoma in profile.

**Figure 5:**
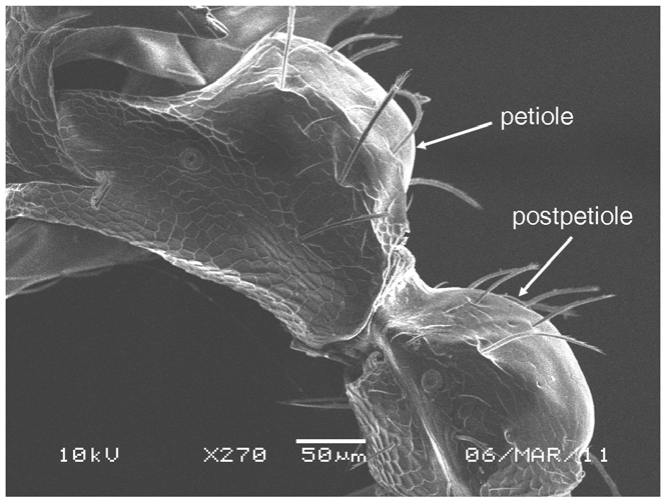
Scanning Electron Micrograph of *Tetramorium amalae* sp. n. holotype worker, petiole and postpetiole in profile.

**Figure 6:**
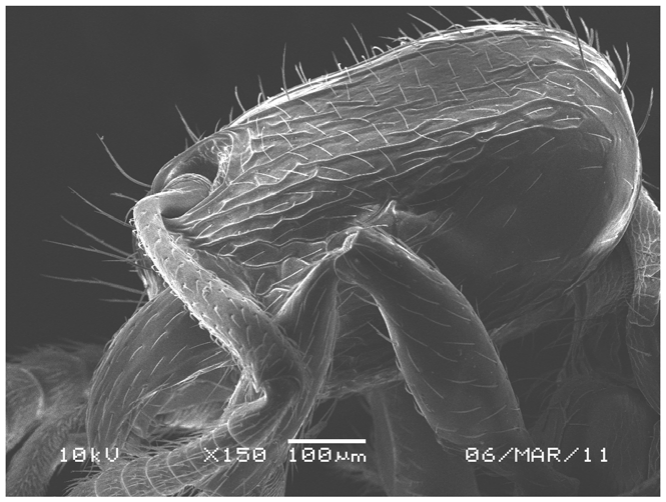
Scanning Electron Micrograph of *Tetramorium amalae* sp. n. holotype worker, head in profile.

**Figure 7:**
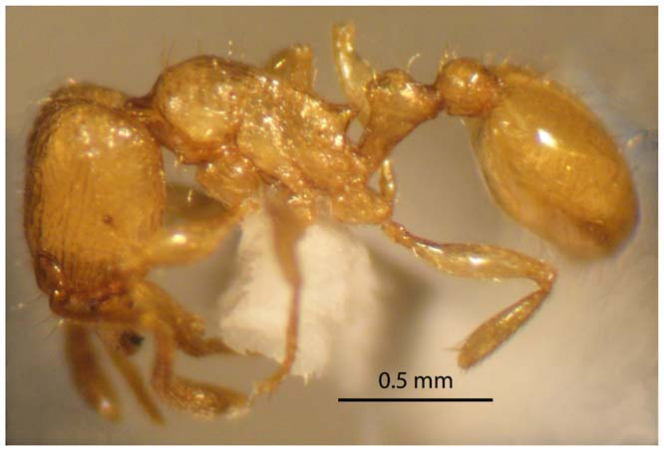
Scanning Electron Micrograph of *Tetramorium amalae* sp. n. holotype worker, body in profile.

**Figure 8:**
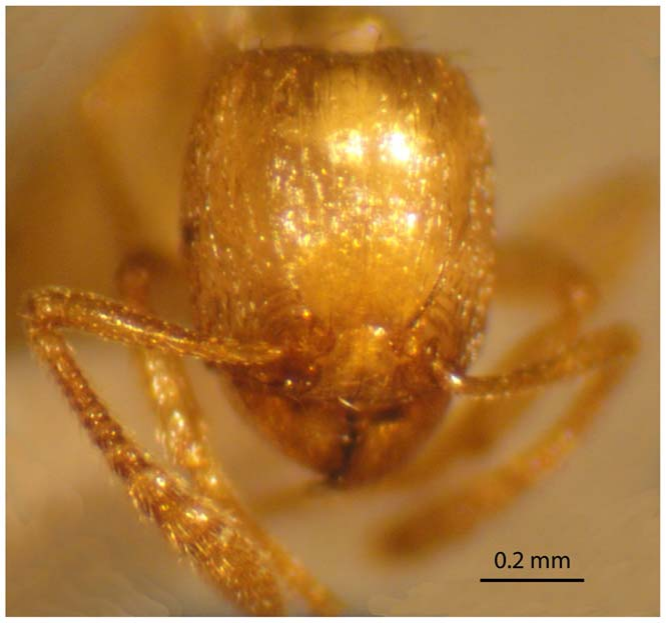
Scanning Electron Micrograph of *Tetramorium amalae* sp. n. holotype worker, head in full-face view.

Saudi Arabia, Al Bahah, Amadan Forest, Al Mandaq, 20.20000 N, 41.21667 E, 1881 m.a.s.l. 19.V.2010 (*M. R. Sharaf & A. S. Aldawood Leg.*). King Saud Museum of Arthropods (KSMA), College of Food and Agricultural Sciences, King Saud University, Riyadh, Kingdom of Saudi Arabia.

#### Paratype worker

Saudi Arabia, Al Bahah,Wadi Turabah, Al Mandaq, 20.21103N,41.28822E, 1739 m.a.s.l. 14.V.2011 (*M. R. Sharaf Leg.*). King Saud Museum of Arthropods (KSMA), College of Food and Agricultural Sciences, King Saud University, Riyadh, Kingdom of Saudi Arabia.

#### Holotype worker

TL 2.55, HL 0.71, HW 0.61, SL 0.44, ML 0.63, PW 0.42, EL 0.01, PL 0.26, PTW 0.15, PPL 0.17, PPW 0.19, SI 72, CI 86.

### Description

Head (Fig. 1) distinctly longer than broad with convex sides and shallowly concave posterior margin. Mandibles (Fig. 1) finely and very faintly longitudinally striated. Anterior clypeal margin with a small notch, the median carina running the length of the clypeus (Fig. 1). Frontal carinae relatively short and weakly developed but distinctly stronger than the other cephalic sculpture, diverging from the frontal lobes and ending at the level of the eyes (Fig. 1). Antennal scrobes visible only as a shallow depression. Eyes tiny (Fig. 2), consisting of only two minute ommatidia on each side, one is smaller than the other and has diameter approximately 0.01, about 0.01×HW and only distinguished under higher magnification. Antennae 12-segmented. Metanotal groove (Fig. 3) feebly impressed. Propodeal spines short and triangular (Fig. 3). Metapleural lobes triangular. Mesosoma sides with irregular wavy longitudinal sculpture (Fig. 4). Propodeal spiracles well developed and circular (Fig. 4). Petiole node rectangular in profile (Fig. 5), with a roughly right-angular anterodorsal angle and oblique posterodorsal angle. In dorsal view the petiole and petiole nodes are distinctly longer than broad, the latter is oblong. Dorsum of head (Figs. 1 and 6) finely but distinctly irregularly longitudinally rugulose, the space between the rugulae finely punctulate. Mesosoma with a faint and low transverse ridge on the anterior pronotum. Promesonotum finely longitudinally rugulose, mesonotum smooth, propodeal dorsum very faintly longitudinally striated. Dorsal surfaces of petiole and postpetiole nodes unsculptured. Gaster smooth and shining. All body surfaces with barbulate numerous fine hairs, the head pilosity is shorter than on the mesosoma and gaster. Colour uniformly yellow.

### Habitat and Biology

**Figure 9:**
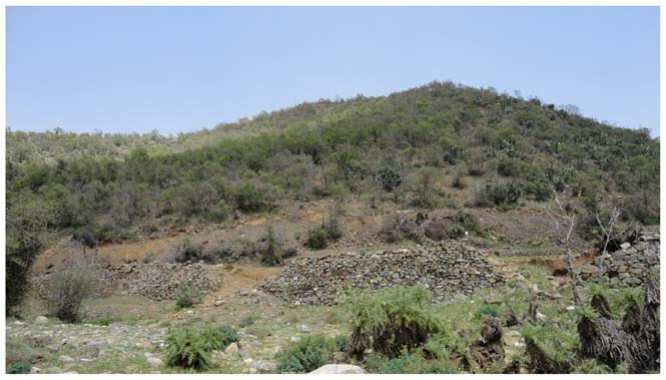
Type locality, Al Bahah,Amadan forest at time of collecting the species.

**Figure 10:**
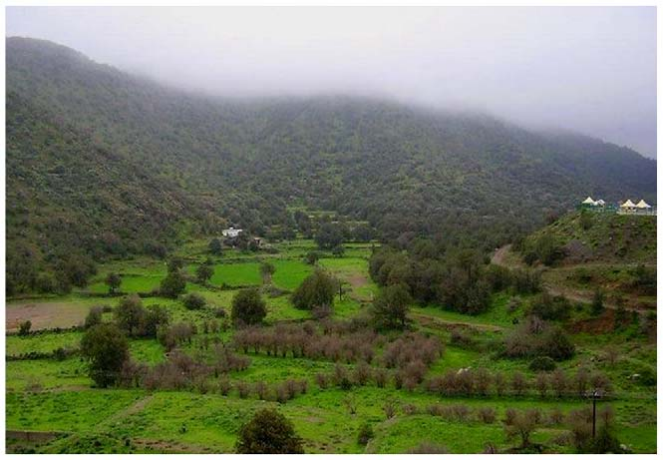
Type locality, Al Bahah,Amadan forest after a season of heavy flooding, from http://travel.maktoob.com/vb/travel450982/ **.**

The type locality (Figs. 9 and 10) is a relatively pristine area. This new species was collected after a season of a relative low rain fall with sparse vegetation cover. It is worth mentioning that in some years heavy rains occur and then usually accompanied by extensive flooding which greatly increases the density of the vegetation (Fig. 10). Nothing is known of the biology of this species. The holotype and the paratype specimens were found in leaf litter samples.

### Etymology

The patronym has been selected to honor both Amal El Saadany (wife of the senior author MRS) and Amal Aldawood (daughter of the second author ASA).

### Diagnosis

*T. amalae* is a member of the *T. shilohense-*group and appears to most resemble *T. dysderke* Bolton [3], described but not illustrated, from Nigeria, in body size and colour but differs in having greater head length, HL 0.71 *versus* 0.59; greater head width, HW 0.61 *versus* 0.50; and, greater pronotal width, PW 0.42 *versus* 0.34; the scape index is smaller, SI 72 *versus* 80 and the eyes are much smaller, EL 0.01×HW *versus* EL 0.06×HW. *T. amalae* has more or less well developed frontal carinae which are stronger than the cephalic sculpture whereas in *T. dysderke* they are very feebly developed and not stronger than the other cephalic sculpture. In addition, in dorsal view the petiole node in *T. amalae* is longer than broad whereas it is about as long as broad in *T. dysderke*. *T. amalae* is also very similar to *T. subcoecum* Forel from Kenya in colour, body measurements and general aspects but they can be separated by the following: antennal scrobes visible only as shallow depression in *T. amalae* whereas no antennal scrobes in *T. subcoecum*; eyes tiny in *T. amalae*, about 0.01×HW, consisting of two ommatidia, whereas in *T. subcoecum* eyes little bit bigger, about 0.04–0.06×HW consisting of a single ommatidia. Another similar but easily distinguishable species is the West African *T. jugatum* Bolton, illustrated by Taylor [22]. Although of a similar size and proportions, that has multi-faceted eyes and more pronounced sculpture on the head and mesosoma [Photographs can be seen on http://antbase.org/ants/africa/tetramorium/tetramorium_jugatum/tetramorium_jugatum.htm]

### Tetramorium latinode Collingwood & Agosti

*Tetramorium latinode* Collingwood & Agosti, 1996: 335, ([19], Fig. 12). Holotype worker, YEMEN: Mabar, pitfall trap, 11.v.1992 *(M. Mahyoub & A. Drews)* (World Museum, Liverpool, England).

#### Materials examined

40 workers, Saudi Arabia, Al Bahah, Amadan forest, Al Mandaq, 20.20000 N, 41.21667 E, 1881 m.a.s.l. 19.V.2010 (*M. R. Sharaf & A. S. Aldawood Leg.*); King Saud Museum of Arthropods (KSMA), College of Food and Agricultural Sciences, King Saud University, Riyadh, Kingdom of Saudi Arabia.

#### Workers

TL 2.62–4.12, HL 0.67–0.80, HW 0.60–0.72, SL 0.42–0.52, ML 0.70–1.00, PW 0.50–0.82, EL 0.12–0.17, PL 0.27–0.40, PTW 0.25–0.35, PPL 0.17–0.22, PPW 0.30–0.42, SI 60–83, CI 87–100 (15 measured).

#### Queen (alate gyne)

TL 3.62, HL 0.77, HW 0.72, SL 0.52, ML 1.10, EL 0.20, PL 0.35, PTW 0.37, PPL 0.25, PPW 0.45, SI 72, CI 94.

#### Alate gyne (not previously described) (Figs. 11, 12)

**Figure 11:**
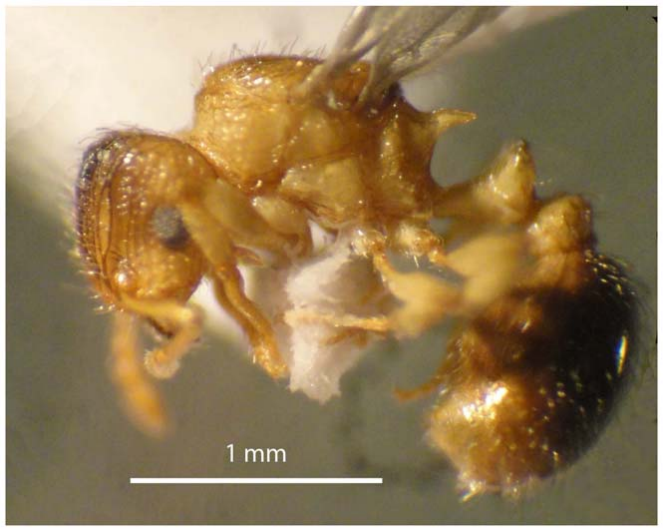
*Tetramorium latinode,* gyne, body in profile.

**Figure 12:**
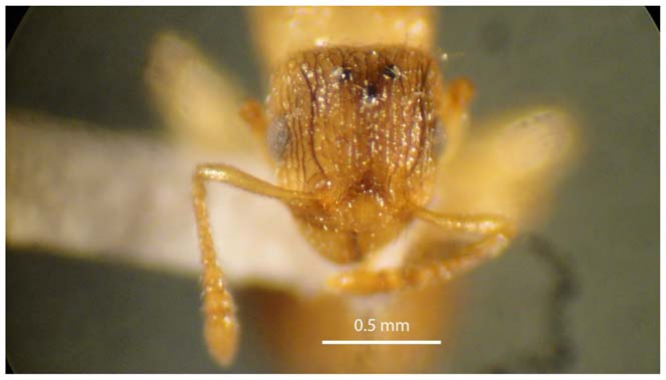
*Tetramorium latinode,* gyne, head in full-face view.

Head little longer than broad with sides nearly straight or feebly convex. Posterior margin of head weakly concave. Eyes large and consist of 14 ommatidia in the longest row, EL 0.27×HW. Antennae 12-segmented. Frontal carinae long and sinuate, reaching back almost to the posterior margin of head where they merge with the remaining sculpture of the cephalic dorsum. Antennal scrobes distinct. Propodeal spines long and acute. Petiole, postpetiole, pilosity and head sculpture are as in worker. Bicoloured, body yellowish, gaster brown.

#### Redescription of Worker (Figs. 13, 14, 15, 16, 17, 18, 19, 20)

**Figure 13:**
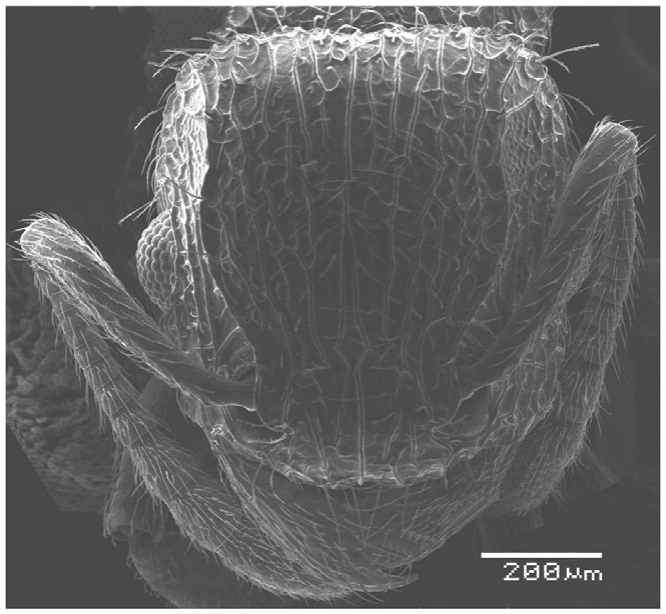
Scanning Electron Micrograph of *Tetramorium latinode,* worker, head in full-face view.

**Figure 14:**
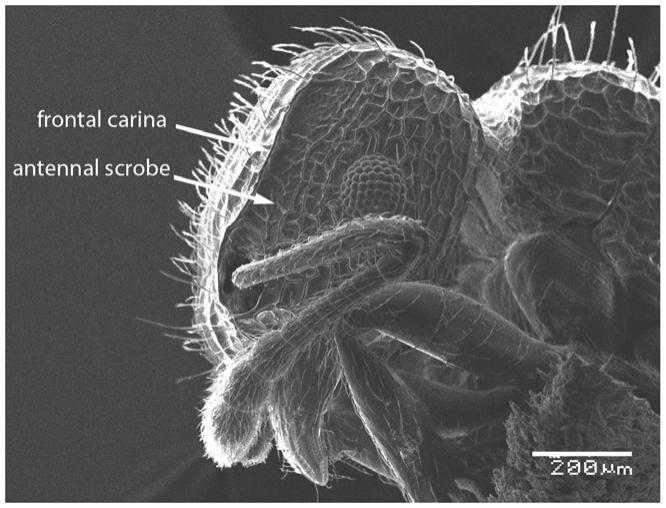
Scanning Electron Micrograph of *Tetramorium latinode,* worker, head in profile.

**Figure 15:**
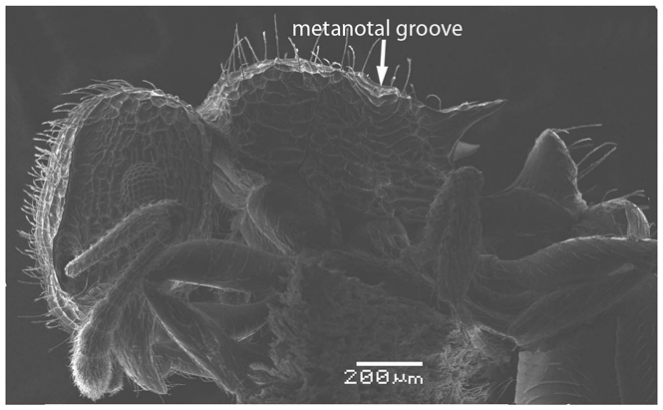
Scanning Electron Micrograph of *Tetramorium latinode,* worker, body in profile.

**Figure 16:**
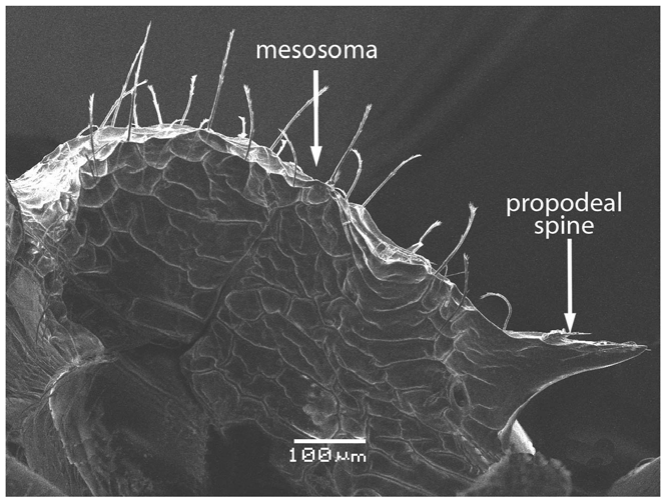
Scanning Electron Micrograph of *Tetramorium latinode,* worker, mesosoma in profile.

**Figure 17:**
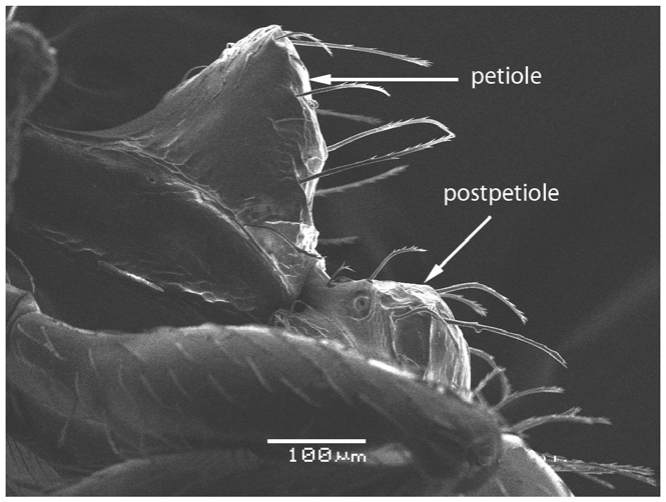
Scanning Electron Micrograph of *Tetramorium latinode,* worker, petiole and postpetiole in profile.

**Figure 18:**
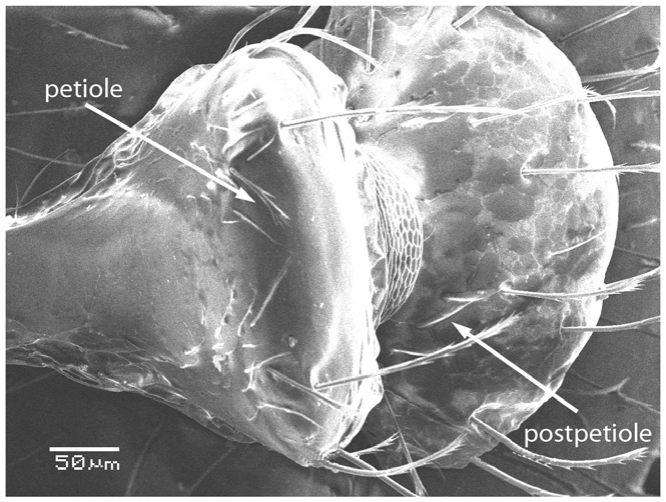
Scanning Electron Micrograph of *Tetramorium latinode,* worker, petiole and postpetiole in dorsal view.

**Figure 19:**
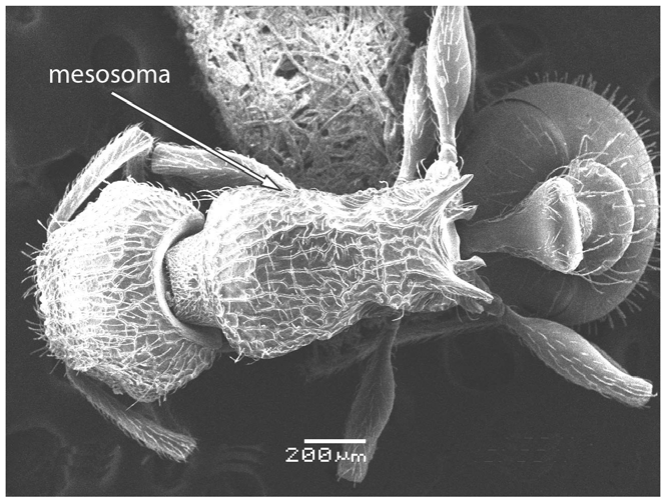
Scanning Electron Micrograph of *Tetramorium latinode,* worker, body in dorsal view.

**Figure 20:**
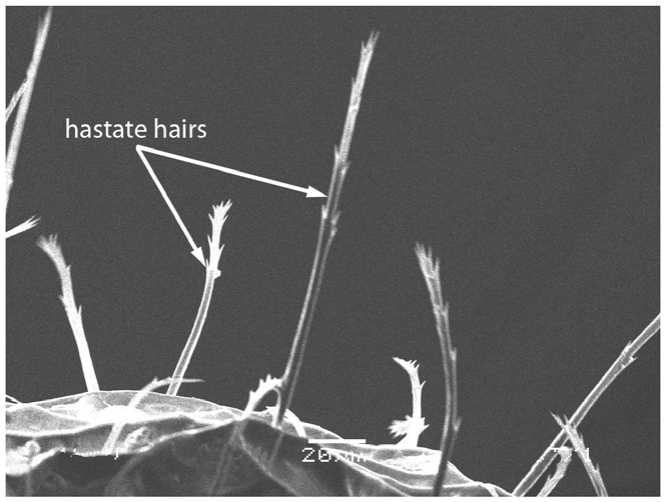
Scanning Electron Micrograph of *Tetramorium latinode,* worker, barbulate hairs.

Head longer than broad with convex sides. Anterior clypeal margin with a median notch or impression. Mandibles faintly longitudinally striated (Fig. 13). Frontal carinae long and sinuate, reaching back almost to the posterior margin of head where they merge with the remaining cephalic dorsum (Figs. 13 and 14). Antennal scrobes distinct (Fig. 14). Eyes large (EL 0.12–0.17) consisting of ten ommatidia in the longest row (Fig. 14).Antennae 12-segmented. Posterior margin of head straight (Fig. 13). Dorsum of mesosoma in profile a continuous curve (Fig. 15). Metanotal groove very feebly impressed (Fig. 15). Propodeal spines elongate and strong, metapleural lobes low and triangular (Fig. 16). Petiole squamiform (Fig. 17), much higher than long in profile and with acute pointed node, in dorsal view (Fig. 18) much broader than long but slightly narrower than the postpetiole which is also clearly broader than long. Postpetiole in profile (Fig. 17) lower than petiole and broadly rounded. Dorsum of head and mesosoma irregularly but quite densely longitudinally rugulose with a reticulum pattern (Fig. 19). Petiole dorsum smooth and shining, postpetiole dorsum more or less smooth and shining with vestiges of patchy pattern (Fig. 18). Gaster smooth and shining. All dorsal surfaces of head and body densely clothed with long, fine, soft finely barbulate hairs (Fig. 20) which are relatively less dense on mesosoma and waist. Antennae and tibiae with dense decumbent pubescence. Colour yellow, the gaster brownish yellow.

### Habitat and Biology

*T. latinode* was originally collected from the Amadan Forest, part of Al Bahah Province (Al Mandaq governorate) about 50 km from Al Bahah to the north. The area is characterized by a substantial degree of endemicity and relatively dense vegetation which differs seasonally depending on rain fall. This vegetation is mainly composed of wild Olive trees, *Acacia*, juniper, and other plants. Our specimens were taken from a nest under a stone on hard-packed soil and close to a large *Juniperus* tree. The nest contained tens of workers and the single alate gyne. The nest was found in relatively elevated area of a valley which is high enough to avoid direct impacts of flooding. No additional nests were found despite extensive surveys. In addition, we were not able to collect foraging workers near the nest.

### Diagnosis

*T. latinode* is a member of the *T. squaminode* group and Collingwood & Agosti [19] suggested close affinities with *T. squaminode*, described from Tanzania. We consider *T. latinode* is more closely related to *T. akermani* Arnold described and illustrated from South Africa [23], [6]. *T. latinode* is yellowish with a brownish yellow gaster, whereas *T. akermani* is dark brown to blackish brown. In addition, the mandibles are faintly longitudinally striated in *T. latinode,* whereas they are smooth and shining in *T. akermani*. *T.latinode* consistently has a smaller head length HL 0.67–0.80 *versus* 0.88–0.94; smaller head width HW 0.60–0.72 *versus* 0.82–0.88, smaller mesosomal length ML 0.70–1.00 *versus* 0.98–1.08 and smaller eye length EL 0.12–0.17 *versus* 0.20–0.21. The queen (Figs. 11 and 12) can be compared with the *T. squaminode* queen shown at http://antbase.org/ants/africa/tetramorium/tetramorium_squaminode/tetramorium_squaminode.htm. Like the worker that has a longer more rectangular head.

### List of Arabian *Tetramorium* species

*bicarinatum* group

 *bicarinatum* (Nylander)

*obesum* group

 *lanuginosum* Mayr

*caespitum* complex

 *biskrense* Forel

 *calidum* Forel

 *chefketi* Forel

 *depressiceps* Menozzi

 *juba* Collingwood

 *syriacum* Emery

*sericeiventre* group

 *khyarum* Bolton

 *sericeiventre* Emery

*setigerum* group

 *doriae* Emery

*shilohense* group

 *amalae* sp. n.

*simillimum* group

 *caldarium* (Roger)

 *delagoense* Forel

 *jizani* Collingwood

 *simillimum* (F. Smith)

 *yemene* Collingwood & Agosti

*squaminode* group

 *latinode* Collingwood & Agosti

“unplaced to group”

 *hirsutum* Collingwood & van Harten

Having read Collingwood & van Harten's description [20] and seen their poor sketch, we find it impossible to place *hirsutum* accurately in a group. It is valid to include it in the list of species as “unplaced to group”.

### A revised key to *Tetramorium* workers of Arabia

1 Body hairs bifid or trifid (cosmopolitan species)………………………………………………………***lanuginosum***

- Body hairs simple or barbulate but not bifid or trifid………2

2 Eyes tiny consisting of only two ommatidia (Saudi Arabia………………………………………………***amalae*** **sp. n.**

- Eyes larger consisting of more than two ommatidia…………3

3 Anterior clypeal margin with a distinct median notch………………………………………………………………4

- Anterior clypeal margin entire, without a median notch………………………………………………………………6

4 Smaller species, TL 2.25–2.37; colour light brown; propodeal spines short and strong (Yemen)……………………………………………………………………………………………***hirsutum***

- Larger species, TL 2.62–4.5; bicoloured species, body distinctly lighter than the dark gaster; propodeal spines long and acute ………………………………….……………………………5

5 Petiole squamiform, much higher than long in profile; body hairs barbulate; head length smaller HL 0.67–0.80; cephalic index greater CI 87–100 (Yemen & Saudi Arabia)……………***latinode***

- Petiole rectangular, not squamiform, distinctly longer than high in profile; body hairs simple; head length greater HL 0.80–1.00; cephalic index smaller CI 80–87 (cosmopolitan invasive species)………………………………………………***bicarinatum***

6 SI 100 or more………………………………………………7

- SI less than 100………………………………………………9

7 Propodeum armed with a pair of strong and well developed spines; lateral portions of clypeus prominent as a tooth or crest on each side in full-face view. When viewed from above and behind the lateral parts of the clypeus arise to a high peak in front of the antennal insertions and then slope steeply down toward the median portion of the clypeus, ***sericeiventre*** **group**……………………8

- Propodeum unarmed, dorsum and declivity merely meeting in an angle, or at most with a pair of minute denticles at the junction of the two surfaces; lateral portion of clypeus not strongly modified as above (Ethiopia, North East Africa, Arabia), ***setigerum*** **group**...................................................................................***doriae***

8 Propodeal dorsum in profile with one or more pairs of hairs arising from the surface between the metanotal groove and the base of the spines (Africa, Saudi Arabia and Yemen)…………………………………………………………………***khyarum***

- Propodeal dorsum in profile without hairs, the posteriormost pair occurring at or before the metanotal groove (Arabia, Africa and the Malagasy region)…………………………***sericeiventre***

9 Hairs on dorsal mesosoma and on first gastral tergite short, stout, and blunt apically, ***simillimum*** **group**…………………10

- Hairs on dorsal mesosoma and first gastral tergite fine and acute apically, or hairs absent from both these surface, ***caespitum*** **complex**………………..…………………………14

10 Frontal carinae extend back to the posterior level of eyes and then are obscured by the cephalic sculpture……………………11

- Frontal carinae extend back beyond the posterior level of the eyes………………………………………………………………12

11 Genae with one long oblique hair, cephalic sculpture more widely spaced, scape index lower with SI 74 (Yemen)……………………………………………………………***yemene***

- Genae with two pairs of hairs, cephalic sculpture closely spaced, scape index higher with SI 83 (Saudi Arabia)………………………………………………………………***jizani***

12 Frontal carinae weakly developed or reduced, either fading out posteriorly or uniformly weak, sometimes broken and usually not more strongly developed than the cephalic sculpture; antennal scrobes very feebly developed or absent; Palp formula 3,2 (a cosmopolitan invasive species)…………………………***caldarium***

- Frontal carinae long and strongly developed throughout their length, running back to the posterior margin of head, the carinae more strongly developed than the cephalic sculpture; antennal scrobes distinct; Palp formula 4,3………………………………13

13 Side of head immediately behind the eyes with a single pair of projecting stout hairs; scapes relatively slightly longer (SI 84–92) (Africa, Malagasy region, Yemen, Palestine)…………***delagoense***

- Side of head immediately behind the eyes without such a hair, either hairless or with a number of fine decumbent to appressed hairs; scapes slightly shorter (SI 74–80) (a cosmopolitan invasive species)……………………….………………………***simillimum***

14 Dorsum of head with a distinct median depressed area (North east Africa and Middle East)………………***depressiceps***

**-** Dorsum of head without a median depression……………15

15 Petiole and postpetiole with distinct dorsal sculpture……16

- Petiole and postpetiole smooth and shining dorsally………17

16 Metanotal groove deep; mesosomal pilosity restricted to pronotum and first half of mesonotum, propodeum bare; scape long (SL 0.78–0.87); petiole and postpetiole nodes coarsely sulcate (Russia & Arabia)…………….……………………………***chefketi***

- Metanotal groove shallow but visible; mesosoma with abundant, stout and relatively long suberect hairs; scape shorter (SL 0.70–0.77); petiole and postpetiole nodes irregularly sculptured (Middle East)……………………………………***syriacum***

17 Head and mesosoma sculpture superficial, smooth in part (Saudi Arabia & Kuwait)………………………………….…***juba***

- Head and mesosoma entirely strongly sculptured…………18

18 Colour black; propodeal spines very short upturned; striae on posterior margin of head divergent (North Africa)…………………………………………………………***biskrense***

- Colour pale reddish yellow; propodeal spines acute and well developed; head striae longitudinal to the posterior margin of head (Oman & Yemen)…………………………………………***calidum***

Note: *T. bicarinatum* is a cosmopolitan invasive species often imported with plant materials. The record from UAE [24] was the first for Arabia and our record, based on a single specimen collected from Riyadh without any specific data, is the first for Saudi Arabia.

## Discussion

With these two species, *T.amalae* and *T. latinode*, the *T. shilohense* group is recorded for the first time in Arabia and the *squaminode* group also is recorded for the first time from Saudi Arabia. *T. latinode* was originally described from Yemen. Both species groups are mainly Afrotropical in distribution [3] with seven and thirteen species for the *shilohense* and *squaminode* groups, respectively. Apparently, these two species are endemic to the chain of Alsarawat Mountains which extends to Yemen. This is supported by the degree of isolation characterizing this area of Arabia.

The finding of these two Afrotropical ant species groups in this area further supports the claim that Al Bahah region in southwestern Arabia biogeographically is Afrotropical [25], [26] and has a distinct history from the rest of the Arabia.

By comparison of the descriptions of *T. jizani* Collingwood and *T. yemene* Collingwood & Agosti, it is apparent also that these two species may be synonymous with the widely distributed invasive species *T.simillimum* (Smith)and *T. delagoense* Forel, as the number of genae hairs overlap. We suspect that the records of *T. khyarum* and *T. delagoense* from Arabia are due to misidentifications but examination of the material mentioned by Collingwood & Agosti [19], and comparison with appropriate type-material will be required.

Finally, the total of nineteen species of *Tetramorium* recorded from Arabia is a relative modest number considering the vastness of the region and the diversity of habitats. We expect additional collecting will reveal additional species.
